# A Preliminary Study of Nutrients Related to the Risk of Relative Energy Deficiency in Sport (RED-S) in Top-Performing Female Amateur Triathletes: Results from a Nutritional Assessment

**DOI:** 10.3390/nu17020208

**Published:** 2025-01-07

**Authors:** Dorota Langa, Marta Naczyk, Robert K. Szymczak, Joanna Karbowska, Zdzislaw Kochan

**Affiliations:** 1 Laboratory of Nutritional Biochemistry, Department of Clinical Nutrition, Medical University of Gdansk, 80-211 Gdansk, Poland; dorota.langa@gumed.edu.pl (D.L.); marta.naczyk@gumed.edu.pl (M.N.); 2 Department of Emergency Medicine, Medical University of Gdansk, 80-214 Gdansk, Poland; robert.szymczak@gumed.edu.pl; 3 Department of Biochemistry, Medical University of Gdansk, 80-211 Gdansk, Poland

**Keywords:** female athletes, triathlon, endurance, sports nutrition, diet, plant protein, fiber, PUFA, energy intake

## Abstract

**Background/Objectives:** As an endurance multi-sport race, triathlon places significant energy demands on athletes during performance and training. Insufficient energy intake from food can lead to low energy availability (LEA) and Relative Energy Deficiency in Sport (RED-S). We aimed to measure symptoms related to LEA, examine the risk of RED-S, and find how diet relates to the risk of RED-S in highly trained female amateur triathletes. **Methods:** Our sample was 20 top-performing female triathletes competing in Quarter Ironman (IM), Half IM, IM, or Double IM triathlons for 5.5 ± 2.5 y who were during the preparatory phase of training (training load 11 ± 3.76 h/week, a single workout 84 ± 25 min). Triathletes completed 3-day food diaries, training diaries, and the Low Energy Availability in Females Questionnaire (LEAF-Q). Exercise energy expenditure was estimated using wrist-worn activity trackers. To examine dietary patterns related to the first signs of LEA, predating RED-S, we created two groups: the L-LEA group (LEAF-Q score 0–5, no symptoms related to LEA, low risk of RED-S, n = 10) and the H-LEA group (LEAF-Q ≥ 6, at least one LEA-related symptom, high risk of RED-S, n = 10). **Results:** The risk of RED-S was prevalent in 30% of female triathletes, and 50% showed at least one symptom related to LEA. Macronutrient intake was similar in all participants, but triathletes from the H-LEA group tended to eat more plant-sourced protein and fiber. They consumed less saturated fatty acids but ingested more significant amounts of *n*-6 polyunsaturated fatty acids (PUFAn6). **Conclusions:** We conclude that foods higher in plant proteins, fiber, and PUFAn6 might predispose female triathletes to LEA by reducing the diet’s energy density.

## 1. Introduction

Relative Energy Deficiency in Sport (RED-S) is a condition that develops due to athletes’ exposure to prolonged or severe low energy availability (LEA). The history of this syndrome dates back to June 1992, when the American College of Sports Medicine (ACSM) convened a consensus conference on the Female Athlete Triad [1]. The term Female Athlete Triad (FAT) was coined to describe a triad of medical disorders, which included disordered eating, amenorrhea, and osteoporosis observed among adolescent and young adult female athletes [1]. In 2014, the International Olympic Committee, recognizing that energy deficiency—an etiological factor for FAT—may also affect men, decided to introduce new terminology: RED-S syndrome was defined as “impaired physiological function including, but not limited to, metabolic rate, menstrual function, bone health, immunity, protein synthesis, cardiovascular health caused by relative energy deficiency” [2]. Athletes may experience varying levels of insufficient energy availability—ranging from adaptable LEA to problematic LEA, the latter being the main reason for long-term impairments of health and performance [3,4].

Mounting evidence suggests that practicing ultra-endurance sports, defined as a series of exercises lasting more than six hours, despite having positive effects, can also negatively impact human health in the long term [5,6,7]. It has not yet been established whether those negative implications apply only to vulnerable individuals or are determined by high training loads and competition demands. As female endurance athletes remain heavily underrepresented in sports science research, the data specific to them are even more limited; however, they suggest important differences in physiological function in men and women in the context of ultra-endurance activity [6,8]. The female characteristics that negatively impact athletic performance include a lower O_2_-carrying capacity and a higher prevalence of gastrointestinal distress [6]. In female endurance athletes, prolonged negative energy balance can lead to menstrual disturbances, predominantly secondary amenorrhea, that might result in temporary infertility, a decline in bone mineral density, and an increased risk of bone stress injuries [9,10,11]. Despite that, the number of females participating in ultra-endurance events is constantly growing, and in recent years, there has been a surge in female involvement with triathlon [5].

Triathlon is a high-intensity sport that consists of three consecutive races of swimming, cycling, and running. As an endurance multi-sport race, triathlon places significant energy demands on athletes during performance and training [6,12,13]. That energy should originate from ingested foods. Insufficient energy intake by endurance athletes can lead to the metabolic state known as LEA [4,5,14]. Energy intake adjusted to maintain normal nutritional status is especially important for female endurance athletes, who are more likely to be at risk for LEA than male athletes [15]. Studies examining dietary patterns related to energy balance and menstrual status in cohorts of female endurance athletes, including triathletes, are still scarce and have yielded conflicting results [16,17,18,19,20]. A study of highly trained runners and triathletes found that energy-deficient amenorrheic athletes consumed 36% less fat but 50% more carbohydrates and 47% more fiber than their eumenorrheic counterparts [16]. In contrast, other studies of amenorrheic and eumenorrheic competitive long-distance runners and triathletes showed no significant differences between groups in nutrient intake [17,18,19]. In a more recent study, researchers reported 26% lower relative fat intake in runners and triathletes with LEA, 23% higher fiber intake in participants with menstrual dysfunction, and no differences between groups in carbohydrate intake [20]. All of these studies involved heterogeneous groups of female endurance athletes, consisting of runners and triathletes, and female triathletes were not distinguished from other study participants [16,17,18,19,20]. Triathlon differs from endurance running in exercise-induced metabolic activity and the requirement for energy substrates—triathletes more than runners rely on lipolysis and fatty acids [21]. Thus, studies on the effect of a diet on energy availability and the risk of RED-S in female triathletes are needed.

This preliminary study aimed to measure symptoms related to LEA, examine the risk of RED-S, and find how diet relates to the risk of RED-S in highly trained female amateur triathletes.

## 2. Materials and Methods

### 2.1. Participants

This study was conducted from December 2022 to March 2023. We selected our sample to include top-performing female triathletes; thus, the inclusion criteria were to place in the top 20 in the open category or the top 10 in the age group category for women in Poland’s most popular, internationally recognized triathlon races such as Ironman, Challenge Family, and Garmin Iron Triathlon. Exclusion criteria were age under 18, BMI < 18.5 kg/m^2^, eating disorders, unstable diabetes, pregnancy, and menopause. To determine the sample size for our research, we used power analysis (alpha = 0.05, beta = 0.2) based on the fiber intake results reported by Melin et al. [20] in a mixed cohort of female endurance athletes that included triathletes with and without LEA symptoms. Power analysis indicated that the minimum sample size needed for adequate study power was 16 (8 + 8) participants.

Participants were recruited individually by sending personalized invitations on Facebook and Instagram. We emailed triathletes detailed information about the research and a standardized consent form. All triathletes gave informed consent to participate in the study. A three-day food diary form, training diary, the Low Energy Availability in Females Questionnaire (LEAF-Q), and written instructions on completing food and training diaries were sent via email. We asked triathletes to report their body mass in kilograms and height in centimeters and to complete a three-day food diary, training diaries, and the LEAF-Q. Participants were encouraged to contact the researchers if they had any questions. The study protocol was approved by the Independent Bioethics Commission for Research of the Medical University of Gdansk (NKBBN/735/2018–2019), on 14 January 2019.

Our study group initially included 22 female amateur triathletes. Triathletes who failed to complete a three-day food diary, training diary, or LEAF-Q were excluded from the study. The final study group consisted of 20 adult women (aged 21–53 years) in the healthy body mass index (BMI) range (18.7–24.9 kg/m^2^) currently practicing for triathlons ≥8 h per week. Female amateur triathletes who participated in this study had been competing in triathlons for an average of 5.5 ± 2.5 years and were racing over different distances: Quarter Ironman (swim 0.95 km, bicycle 45 km, run 10.5 km), Half Ironman (Triathlon 70.3; swim 1.9 km, bicycle 90 km, run 21 km), Ironman (Triathlon; swim 3.9 km, bicycle 181 km, run 42 km), Double Ironman (swim 7.6 km, bicycle 360 km, run 84.4 km). Athletes were during the preparatory phase of training, and their training programs included swimming, cycling, running, stretching, and strength conditioning exercises. The training sessions occurred daily, except for three participants (15, 17, and 18), with one recovery day. The average self-reported training load was 11 ± 3.76 h/week, with an average single workout estimated using wrist-worn activity trackers lasting 84 ± 25 min. Table 1 presents the baseline characteristics of participants.

### 2.2. Dietary Assessment

We assessed triathletes’ dietary intakes using their records of the amounts of foods and beverages consumed over three consecutive days. To help female triathletes estimate the amounts of food eaten, we provided them access to the album of photographs of food products and dishes published by the National Food and Nutrition Institute (Poland) [22] and a household measurement conversion chart. A total of 8 out of 20 participants completed a food diary with accuracy to grams; the rest of the study group used household measures (8 participants) or both methods simultaneously (4 participants). A certified dietitian (D.L.) reviewed the reports with the participants at the end of the recording period.

### 2.3. Resting Energy Expenditure

Resting energy expenditure (REE) was calculated using the Harris–Benedict equation revised by Roza and Shizgal [23]:

REE = 9.247 × body mass (kg) + 3.098 × height (cm) − 4.330 × age (years) + 447.593.(1)

### 2.4. Exercise Energy Expenditure

Exercise energy expenditure (EEE) was estimated using wrist-worn activity trackers such as Garmin (18 participants), Suunto (one participant), and Polar (one participant).

### 2.5. Physical Activity Level, Total Energy Expenditure

Female amateur triathletes who participated in this study have sedentary jobs; thus, the physical activity level (PAL) of 1.4 was used, as recommended by the FAO/WHO/UNU Experts [24] for a sedentary or light activity lifestyle, in equations to determine participants’ total energy expenditure (TEE). Values of EEE from wrist-worn devices were added. Energy intake (EI), TEE, and energy deficit (ED) are presented as absolute values (kcal/d) and body mass-adjusted relative values (kcal/kg/d). Total energy expenditure was calculated as follows:

TEE = REE × 1.4 + EEE.(2)

### 2.6. Low Energy Availability in Females Questionnaire

All triathletes participating in our study completed the LEAF-Q, which consisted of 25 questions divided into 3 sections—concerning injuries, gastrointestinal symptoms, and menstrual dysfunction [14].

The LEAF-Q is a questionnaire validated in female endurance athletes, including triathletes, that measures symptoms related to LEA and can be used to identify female athletes at risk of RED-S [14]. The authors of the LEAF questionnaire proposed the following cut-off scores: ≥2 for injuries, ≥2 for gastrointestinal symptoms, and ≥4 for menstrual dysfunction [14]. Using these cut-offs, they categorized female athletes with a total score of ≥8 as at risk of RED-S, while those with a total score of <8 had a low risk of RED-S [14]. Later studies involving controlled caloric restriction and exercise intervention in young women demonstrated that varying levels of energy deficiency can induce menstrual disturbances in a dose-dependent manner and that energy availability is a significant predictor of menstrual cycle disturbances; however, no threshold of energy availability induces these disturbances [9,10].

Thus, we initially used a threshold of 8 pts and above to classify participants as at risk of RED-S. However, later, in order to detect the very first signs of LEA that may predate RED-S and examine associated dietary patterns, we assigned female triathletes participating in our study to one of two groups based on a total LEAF-Q score: the low LEA (L-LEA) group, which consisted of triathletes who scored 0–5 pts in the LEAF-Q and exhibited no symptoms related to LEA—we classified them as having low risk of RED-S; and the high LEA (H-LEA) group, which consisted of triathletes who scored ≥ 6 pts and exhibited at least one symptom related to LEA—we classified them as being at high risk of RED-S.

### 2.7. Statistical Analysis

Three-day diet records were analyzed by certified dietitians (D.L. and M.N.) using the Kcalmar.pro software (Hermax, Lublin, Poland). Kcalmar.pro, in addition to the UK “Composition of foods integrated dataset”, the Swiss Food Composition Database, and the USDA Database, includes “Tables of composition and nutritional value of food” [25], developed by the Polish National Institute of Public Health–National Institute of Hygiene and containing foods that best represent those consumed by the participants of our study.

We used the Shapiro–Wilk test to assess the normality of the data and Levene’s test to assess the homogeneity of variances. The t-Student and Mann–Whitney U tests were used to compare differences between groups. Effect sizes were calculated using Cohen’s d test to quantify the magnitude of differences between groups; 95% confidence intervals (CI) were provided to assess the precision of these estimates. We used Fisher’s exact test to determine whether the prevalence of omnivores and various types of vegetarians differs between groups. Quantitative data’s mean and standard deviation (SD) values were presented. *p* < 0.05 was considered as a significant difference. Statistical analysis was performed using the R software (version 4.4.1).

## 3. Results

### 3.1. Low Energy Availability in Females Questionnaire Scores

**Figure 1:**
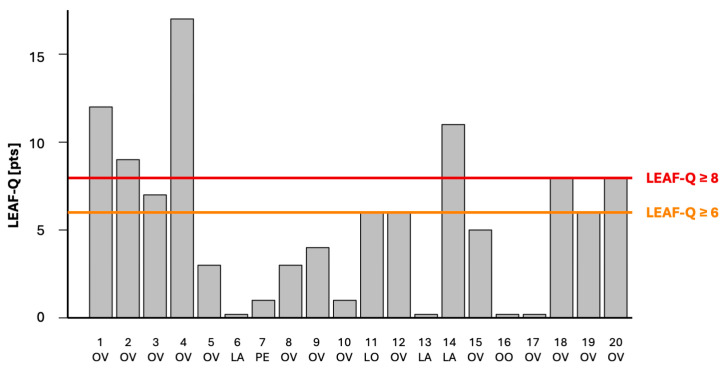
Low Energy Availability in Females Questionnaire (LEAF-Q) scores in top-performing female amateur triathletes. Triathletes with LEAF-Q scores ≥ 8 were classified as at risk of Relative Energy Deficiency in Sport (RED-S). Triathletes who scored ≥ 6 and exhibited at least one symptom related to LEA were deemed at high risk of RED-S and assigned to the H-LEA group. Diet: LA, lacto-vegetarian; LO, lacto-ovo-vegetarian; OO, ovo-vegetarian; OV, omnivorous; PE, pescatarian.

Total LEAF-Q scores in top-performing female amateur triathletes participating in our study ranged from 0 to 17 pts (Table 2, Figure 1). Based on the scoring and the cut-offs proposed previously by the authors of the LEAF questionnaire [14], initially, we divided the cohort into two categories: a LEAF-Q score ≥ 8 pts, indicating risk for RED-S, and a LEAF-Q score < 8 pts, indicating low risk for RED-S. In our study, the risk of RED-S was prevalent in 30% of triathletes—6 out of 20 scored ≥ 8 pts (Figure 1). Fourteen triathletes, i.e., 70%, had LEAF-Q scores of < 8 pts and were classified as having a low risk of RED-S.

Next, exploring the very first signs of LEA that may predate RED-S, we found that 10 out of 20 female triathletes exhibited no symptoms related to LEA—they had a total LEAF-Q score of 0–5 pts (Table 2, Figure 1). Thus, 50% of our study participants, not having any LEA symptoms, had a low risk of RED-S; thus, we assigned them to the L-LEA group (n = 10). Ten other triathletes, i.e., 50%, scored ≥ 6 pts and exhibited at least one symptom related to LEA; we classified them as at high risk of RED-S and assigned them to the H-LEA group (n = 10). In the H-LEA group, 8 out of 10 triathletes exhibited menstrual dysfunction, 3 out of 10 had gastrointestinal symptoms, and 2 out of 10 had injuries (Table 2).

### 3.2. Energy Assessment

**Figure 2:**
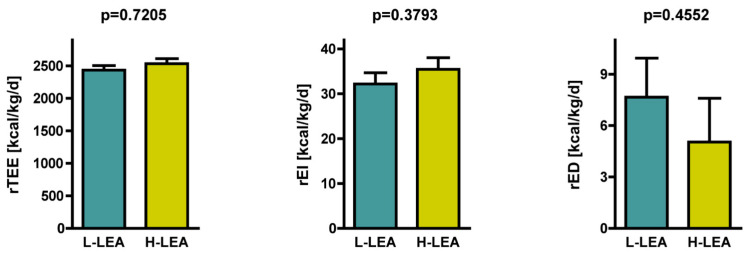
Relative total energy expenditure (rTEE), relative energy intake (rEI), and relative energy deficit (rED) in female amateur triathletes. Relative energy deficit is shown in absolute values. L-LEA, low LEA (LEAF-Q score 0–5 pts); H-LEA, high LEA (LEAF-Q score ≥ 6 pts).

The absolute total energy expenditure (aTEE) and relative total energy expenditure (rTEE) did not differ significantly between groups (Table 3, Figure 2). The absolute energy intake (aEI) and relative energy intake (rEI) tended to be higher in the H-LEA group than in the L-LEA group. We found that 16 out of 20 female amateur triathletes experienced energy deficits. The absolute energy deficit (aED) and relative energy deficit (rED) tended to be lower in the H-LEA group than in the L-LEA group.

### 3.3. Training Load

The average self-reported training load was lower in female triathletes from the H-LEA group (9.5 ± 1.35 h/week) than in those from the L-LEA group (12.5 ± 4.79 h/week); *p* = 0.0374 (Table 4). There were no significant differences in the longevity of the training sessions estimated using wrist-worn activity trackers during the study period between the L-LEA and H-LEA groups (88.4 ± 33.5 vs. 80.2 ± 14.3 min/d, respectively; *p* = 0.7620).

### 3.4. Dietary Intake of Macronutrients

**Figure 3:**
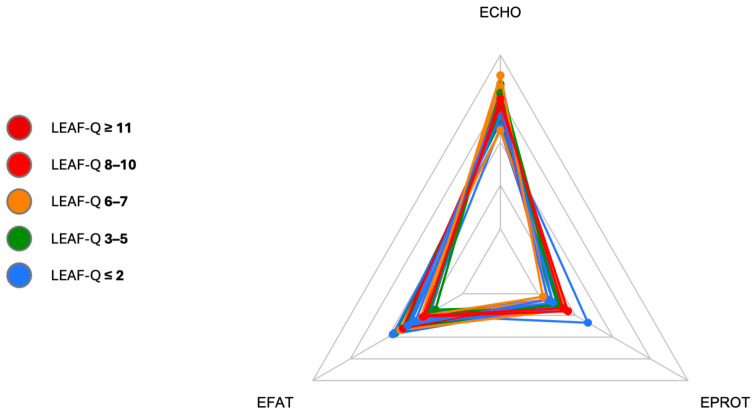
Energy intake from macronutrients. ECHO, energy intake from carbohydrates [%]; EPROT, energy intake from proteins [%]; EFAT, energy intake from fats [%]; LEAF-Q, Low Energy Availability in Females Questionnaire. All study participants are displayed in one radar plot with different colors allocated to various LEAF-Q scores: LEAF-Q score ≤ 2 pts (blue), LEAF-Q score 3–5 pts (green), LEAF-Q score 6–7 pts (orange), LEAF-Q score 8–10 pts (red), and LEAF-Q score ≥ 11 pts (dark red). Each polygon represents one participant.

Energy-adjusted macronutrient intake was similar among all study participants (Figure 3). In female triathletes, carbohydrates accounted, on average, for about 53% of daily energy, protein for about 18%, and fat for about 29% of the energy from the diet. The proportions of participants on an omnivorous vs. vegetarian diet did not differ significantly between the L-LEA and H-LEA groups (Fisher’s exact test; *p* = 0.6285).

#### 3.4.1. Carbohydrates

There were no significant differences in the dietary intake of carbohydrates between groups. Triathletes from the L-LEA group consumed, on average, 260.5 ± 61.9 g of carbohydrates daily, while those from the H-LEA group 313.4 ± 74.5 g/d (*p* = 0.1014). Adjusted by body mass, relative carbohydrate intake was 4.3 ± 1.3 g/kg/d in the L-LEA group and 5.1 ± 1.4 g/kg/d in the H-LEA group (*p* = 0.2400). The intake of energy from carbohydrates was similar in both groups—carbohydrates provided 52.4 ± 6.5% of daily energy in the L-LEA group and 54.2 ± 5.4% in the H-LEA group (*p* = 0.5100).

#### 3.4.2. Protein

**Figure 4:**
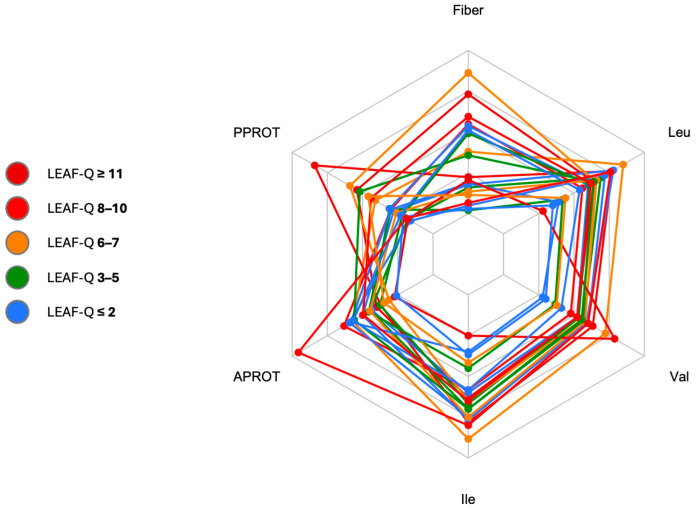
Daily intake of fiber (fiber) [g/d], leucine (Leu) [g/d], valine (Val) [g/d], isoleucine (Ile) [g/d], proteins of animal origin (APROT) [g/kg/d], and proteins of plant origin (PPROT) [g/kg/d]. LEAF-Q, Low Energy Availability in Females Questionnaire. All study participants are displayed in one radar plot with different colors allocated to various LEAF-Q scores: LEAF-Q score ≤ 2 pts (blue), LEAF-Q score 3–5 pts (green), LEAF-Q score 6–7 pts (orange), LEAF-Q score 8–10 pts (red), and LEAF-Q score ≥ 11 pts (dark red). Each polygon represents one participant.

Mean protein intake did not differ significantly between groups for either total (88.8 ± 13.5 g/d in the L-LEA group vs. 100.9 ± 25.0 g/d in the H-LEA group; *p* = 0.1954) or body mass-adjusted relative amounts (1.47 ± 0.31 g/kg/d in the L-LEA group vs. 1.62 ± 0.45 g/kg/d in the H-LEA group; *p* = 0.3845). Dietary protein accounted for 18.4 ± 4.7% of daily energy in the L-LEA group and 17.6 ± 3.5% in the H-LEA group; *p* = 0.6631. Dietary intake of branched-chain amino acids was also similar in both groups (Figure 4).

**Figure 5:**
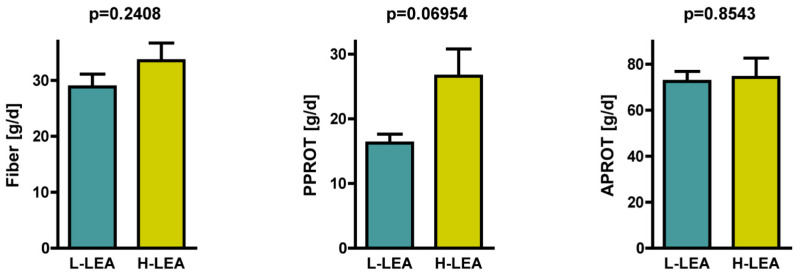
Daily intake of fiber [g/d], proteins of plant origin (PPROT) [g/d], and proteins of animal origin (APROT) [g/d]. L-LEA, low LEA (LEAF-Q score 0–5 pts); H-LEA, high LEA (LEAF-Q score ≥ 6 pts).

There were no significant differences in the intake of animal-sourced protein: 72.5 ± 13.7 g/d in the L-LEA group vs. 74.3 ± 26.4 g/d in the H-LEA group; *p* = 0.8543 (Figure 5). However, female triathletes from the H-LEA group tended to consume more plant-sourced protein (26.6 ± 13.3 g/d) than those from the L-LEA group (16.2 ± 4.4 g/d); *p* = 0.05627 (Cohen’s d = 1.04, large) (Figure 5, Table 5).

#### 3.4.3. Fat

Total fat intake was similar in both groups—female triathletes from the L-LEA group consumed, on average, 64.8 ± 19.4 g of fat daily, while those from the H-LEA group consumed 71.1 ± 13.8 g/d (*p* = 0.4115). It accounted for 29.2 ± 5.8% of energy intake from fat in the L-LEA group and 28.2 ± 4.6% in the H-LEA group (*p* = 0.6794). The relative fat intake was also similar in the L-LEA group (1.06 ± 0.31 g/kg/d) and in the H-LEA group (1.15 ± 0.26 g/kg/d); *p* = 0.5382.

**Figure 6:**
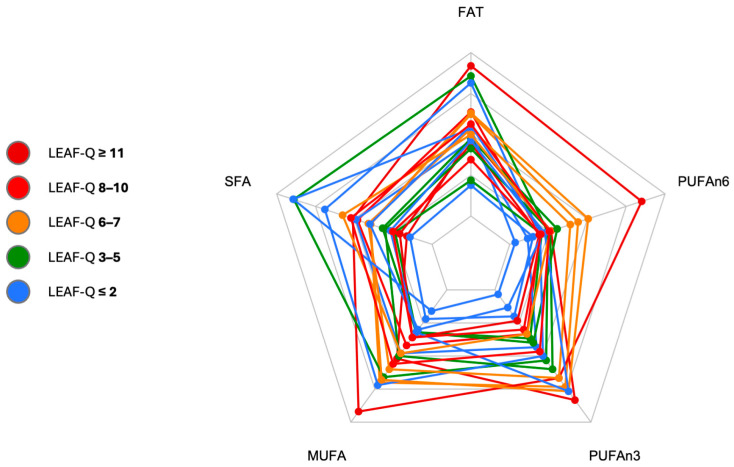
Daily intake of fat (FAT) [g/d], *n*-6 polyunsaturated fatty acids (PUFAn6) [g/d], *n*-3 polyunsaturated fatty acids (PUFAn3) [g/d], monounsaturated fatty acids (MUFA) [g/d], and saturated fatty acids (SFA) [g/d]. LEAF-Q, Low Energy Availability in Females Questionnaire. All study participants are displayed in one radar plot with different colors allocated to various LEAF-Q scores: LEAF-Q score ≤ 2 pts (blue), LEAF-Q score 3–5 pts (green), LEAF-Q score 6–7 pts (orange), LEAF-Q score 8–10 pts (red), and LEAF-Q score ≥ 11 pts (dark red). Each polygon represents one participant.

**Figure 7:**
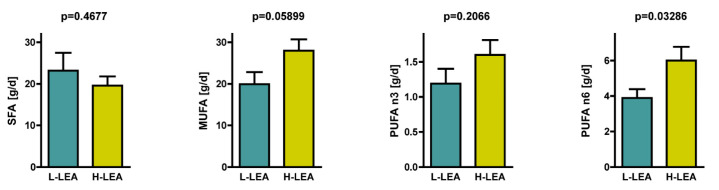
Daily intake of saturated fatty acids (SFA) [g/d], monounsaturated fatty acids (MUFA) [g/d], *n*-3 polyunsaturated fatty acids (PUFAn3) [g/d], and *n*-6 polyunsaturated fatty acids (PUFAn6) [g/d]. L-LEA, low LEA (LEA 0–5); H-LEA, high LEA (LEA ≥ 6).

**Figure 8:**
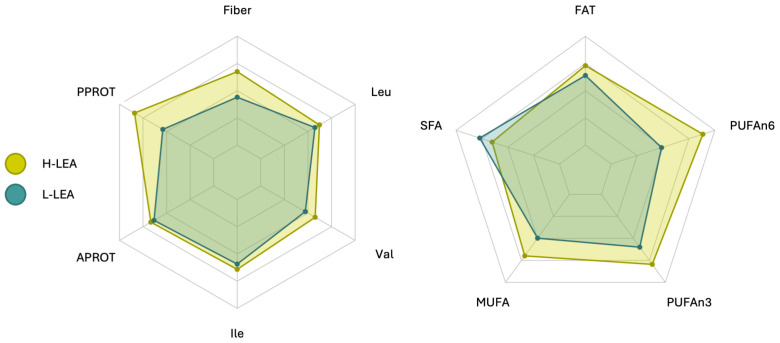
Daily intake of fiber (fiber) [g/d], leucine (Leu) [g/d], valine (Val) [g/d], isoleucine (Ile) [g/d], proteins of animal origin (APROT) [g/kg/d], proteins of plant origin (PPROT) [g/kg/d], fat (FAT) [g/d], *n*-6 polyunsaturated fatty acids (PUFAn6) [g/d], *n*-3 polyunsaturated fatty acids (PUFAn3) [g/d], monounsaturated fatty acids (MUFA) [g/d], and saturated fatty acids (SFA) [g/d]. Two groups (L-LEA and H-LEA) are displayed in each radar plot with two different colors. L-LEA, low LEA (LEAF-Q score 0–5 pts); H-LEA, high LEA (LEAF-Q score ≥ 6 pts).

However, the amounts of saturated fatty acids (SFA), monounsaturated fatty acids (MUFA), and polyunsaturated fatty acids (PUFA) in the diet and their relation to each other varied between groups (Figure 6). Triathletes from the L-LEA group tended to ingest more SFA (23.2 ± 13.6 g/d) than those from the H-LEA group (19.6 ± 7.1 g/d); *p* = 0.4677 (Cohen’s d = −0.33, small) (Table 5, Figure 7), but participants from the H-LEA group tended to consume more unsaturated fatty acids than those from the L-LEA group: 28.0 ± 8.6 g/d vs. 19.9 ± 9.2 g/d of MUFA (*p* = 0.05899; Cohen’s d = 0.90, large), and 1.6 ± 0.7 g/d vs. 1.2 ± 0.7 g/d of *n*-3 polyunsaturated fatty acids (PUFAn3) (*p* = 0.2066; Cohen’s d = 0.59, medium). Furthermore, the dietary intake of *n*-6 polyunsaturated fatty acids (PUFAn6) was significantly higher in female triathletes from the H-LEA group (7.1 ± 4.1 g/d) than in those from the L-LEA group (3.9 ± 1.5 g/d); *p* = 0.03286 (Cohen’s d = 1.02, large) (Figure 7 and Figure 8).

### 3.5. Fiber Intake

Dietary fiber intake by female triathletes participating in our study ranged from 20.7 to 50.2 g daily (Figure 4). Participants from the H-LEA group tended to consume more fiber than those from the L-LEA group. Fiber intake was 33.5 ± 9.9 g/d on average in triathletes from the H-LEA group and 28.8 ± 7.2 g/d in the L-LEA group; *p* = 0.2408 (Cohen’s d = 0.54, medium) (Table 5, Figure 5 and Figure 8).

### 3.6. Dietary Intake of Micronutrients—Iron and Calcium

Dietary intake of iron by female amateur triathletes ranged from 8.8 to 25.3 mg/d. Triathletes from the L-LEA group had lower iron intake than those from the H-LEA group—they consumed, on average, 13.6 ± 2.7 mg of iron daily, while those from the H-LEA group consumed 16.4 ± 5.4 mg/d (*p* = 0.3843).

Triathletes participating in our study consumed 392.8 to 1776.7 mg of calcium daily. The dietary intake of this micronutrient was lower in the L-LEA group (848.9 ± 444.6 mg/d) than in the H-LEA group (1014.3 ± 249.6 mg/d), *p* = 0.2176.

## 4. Discussion

This study’s main finding is that dietary patterns higher in unsaturated fatty acids, plant proteins, and fiber might increase RED-S risk in highly trained, top-performing female amateur triathletes. RED-S syndrome results from metabolic adaptations to low energy availability (LEA) and affects all body systems [3,4,6]. Our study used the LEAF-Q, a questionnaire that collects data on injuries, gastrointestinal symptoms, and menstrual dysfunction, and was validated in female endurance athletes [14] to identify participants with symptoms related to LEA—and thus at risk of RED-S—among top-performing female amateur triathletes.

Initially, we used the scoring and cut-offs proposed by the authors of the LEAF questionnaire [14], classifying those of our triathletes who scored ≥ 8 pts as at risk of RED-S and those who scored < 8 pts as having a low risk of this syndrome. We found that 6 of 20 female triathletes participating in our study (i.e., 30%) had LEAF-Q scores ≥ 8. This percentage is lower than reported by Melin et al. [14] in the mixed cohort of female endurance athletes, including dancers, long-distance runners, and triathletes. In their study, 28 out of 45 athletes, i.e., 62%, had LEAF-Q scores ≥ 8, but there were no data concerning just triathletes. The high prevalence of underweight athletes (six subjects with a BMI of < 18.5 kg/m^2^) and those with eating disorders (eleven subjects) in their cohort are probably the reasons for the high number of athletes at risk of RED-S. In our study, a BMI of < 18.5 kg/m^2^ and eating disorders were exclusion criteria. On the other hand, Witkoś et al. [26], conducting a study on the incidence of injuries, gastrointestinal problems, and menstrual disturbances in female triathletes, found that 10% of them (3 out of 30) had a LEAF-Q score ≥ 8, which is lower than our findings. However, female triathletes participating in their study had a lower training load (6.7 ± 1.6 h/week) than the self-reported training load of top-performing female triathletes who participated in our research (11 ± 3.76 h/week).

In our study, 80% of triathletes experienced energy deficits. Evidence indicates that an energy deficit resulting from exercise and dietary restriction induces menstrual disturbances in a dose-dependent manner [10]. Energy availability is a significant predictor of menstrual disturbances—they increase linearly as energy availability decreases, so there is no energy availability threshold below which menstrual disturbances are induced [9]. Prolonged insufficient energy availability is a leading cause of RED-S [3,4]. Thus, in order to explore dietary patterns in triathletes exhibiting the very first signs of LEA, predating RED-S, we used LEAF-Q scores to split our cohort into two groups: the low LEA (L-LEA) group, which consisted of triathletes who had a total LEAF-Q score 0–5 pts, showed no symptoms related to LEA, and thus had a low risk of RED-S; and the high LEA (H-LEA) group, which consisted of triathletes who scored ≥ 6 pts in the LEAF-Q, showed at least one symptom related to LEA, and, thus, were at high risk of RED-S. Among triathletes with LEA symptoms (in the H-LEA group), the most prevalent were those exhibiting menstrual dysfunction (8 out of 10), 3 out of 10 had gastrointestinal symptoms, and 2 out of 10 had injuries.

The energy-adjusted dietary intake of primary macronutrients was similar among female triathletes participating in our research. On average, carbohydrates accounted for about 53% of the energy from the diet, protein for about 18%, and fat for about 29% of ingested energy. The novel finding of our study is that diets consumed by triathletes exhibiting symptoms related to LEA differed in protein type (more plant-sourced than animal-sourced) and proportion of fatty acids (more unsaturated than saturated) from diets of triathletes without LEA symptoms.

We observed that the average dietary intake of carbohydrates did not differ between the L-LEA and H-LEA groups. Our findings agree with previous studies showing no differences in carbohydrate energy intake between endurance-trained eumenorrheic runners and triathletes and their amenorrheic, exhibiting symptoms related to LEA counterparts [17,18,19,20]. By contrast, Laughlin and Yen [16] found that highly trained female runners and triathletes with menstrual disturbances showed significantly higher carbohydrate intake (70.2 ± 3.4% of energy) than eumenorrheic athletes (53.8 ± 4.8% of energy). Carbohydrates are easily digested, absorbed, and metabolized; thus, they are the primary fuel source for high exercise and athletic performance [27]. They are also required for post-exercise muscle glycogen resynthesis and rapid repletion of glycogen stores in the liver [28]. Recommendations for carbohydrate intake vary depending on the demands of training and competition [29]. In our study, triathletes from both groups achieved a carbohydrate intake recommended for the general population, ranging from 3 to 10 g/kg of body mass daily [29]. However, they failed to meet current sports nutrition guidelines, which recommend carbohydrate intake of 6–10 g/kg/d for athletes engaged in endurance training, i.e., 1–3 h of moderate- to high-intensity exercise daily [29].

Our study demonstrated that female triathletes from the L-LEA group consumed 1.47 g of protein per kg of body mass daily, and those from the H-LEA group consumed 1.62 g/kg/d. However, the diet of the participants at risk of RED-S contained more protein from plants than that of the athletes with a low risk of RED-S, not exhibiting symptoms related to LEA. The current dietary recommendations for protein intake in endurance athletes range from 1.2 to 2.0 g/kg/d [29]. Thus, the study participants met the dietary recommendations for protein intake. A similar dietary protein intake, i.e., about 1.6 g/kg/d, was reported in adult female triathletes who participated in the first stage of the Brazilian Short Triathlon [30]. A study of female amateur cyclists and triathletes found that a dietary protein intake of 1.63 g/kg/d is required to maintain nitrogen balance during training of moderate intensity and duration [31]. Protein requirements are increased in athletes to provide amino acids for the repair and remodeling of body proteins during training adaptations and to replace amino acids lost during exercise-induced oxidation [32]. Dietary protein ingestion and increased plasma amino acid levels stimulate muscle protein synthesis [33]. Proteins can be sourced from animal and plant foods, but plant-based foods are less protein-dense than animal-based food sources [34]. The digestion and absorption of proteins from plants are also less effective when compared with animal proteins—probably because of the presence of fiber and other non-starch polysaccharides as well as antinutritional factors such as hemagglutinins and tannins that decrease the digestibility of plant proteins and bioavailability of amino acids [35,36]. Moreover, plant proteins usually contain lower amounts of essential amino acids than proteins of animal origin and thus have a less optimal amino acid composition and lower anabolic potential [36].

In this study, the total fat and fat energy intake were similar among all participants. Our findings are in line with earlier studies that observed comparable fat energy intake in endurance-trained female runners and triathletes with normal menstrual function and those with menstrual disorders [18,19,20]. Few previous studies have observed lower dietary fat intake in highly trained amenorrheic runners and triathletes than in their eumenorrheic counterparts [16,17]. A study by Melin et al. [20] involving a mixed cohort of female endurance athletes that included triathletes found that subjects without symptoms related to LEA had significantly higher relative fat intake (1.9 ± 0.6 g/kg/d) than those with LEA (1.4 ± 0.2 g/kg/d). In contrast, we did not observe any differences in the relative fat intake between the L-LEA and H-LEA groups; the difference was in fat composition.

A novel finding from our study was that the proportion of saturated and unsaturated fatty acids ingested differed between groups. Female triathletes from the L-LEA group, i.e., without symptoms related to LEA, tended to consume more saturated fatty acids (SFA) than triathletes at risk of RED-S (from the H-LEA group), who tended to ingest more unsaturated fatty acids. Specifically, the dietary intake of *n*-6 polyunsaturated fatty acids (PUFAn6) was significantly higher in the H-LEA group than in the L-LEA group. Research indicates that PUFAn6 are very efficient activators of human uncoupling proteins UCP2 and UCP3, which are particularly abundant in skeletal muscles [37,38]. Activation of UCP3 by PUFAn6 may promote fatty acid oxidation and energy dissipation through uncoupling activity [38]. Since the primary source of PUFAn6 is plant foods, these results suggest that triathletes exhibiting LEA symptoms consumed a diet with lower energy density than their counterparts from the L-LEA group. Dietary patterns high in unsaturated fatty acids are usually rich in vegetables, fruits, legumes, and whole grains. An increased proportion of these food components might reduce the energy density of the diet. Evidence indicates that energy-dense dietary patterns are high in SFA [39]. The results of our study show that dietary fat composition is vital for triathletes who might benefit from higher dietary SFA intake.

This study also revealed that female amateur triathletes at risk of RED-S (from the H-LEA group) tended to eat more fiber than those from the L-LEA group. To our knowledge, this is the first study investigating dietary fiber intake and symptoms related to LEA in female triathletes. A few studies have explored the effects of fiber in female endurance athletes, including triathletes, but none have distinguished them from other athletes [16,20]. A study by Laughlin and Yen [16] involving highly trained female runners and triathletes showed that amenorrheic athletes consumed 47% more fiber than their eumenorrheic counterparts. Similar findings were reported by Melin et al. [20] in the mixed cohort of female endurance athletes—the athletes with functional hypothalamic oligomenorrhea/amenorrhea and LEA ate more fiber than those with regular menstrual cycles and optimal energy availability. Fiber is an important part of a healthy diet; however, high fiber intake can result in nutrient deficiencies [40]. Dietary fiber might suppress energy intake by inducing satiety, delaying gastric emptying, and reducing ingestion [40,41]. Moreover, increased dietary fiber intake might impair intestinal absorption of macronutrients—carbohydrates, proteins, and fats—which serve as a body energy source. It might also adversely affect the absorption of micronutrients such as water-soluble vitamins and minerals, including iron and calcium.

Iron is an essential component of oxygen-carrying hemoglobin, myoglobin, and many mitochondrial proteins involved in energy production [42]. The recommended dietary allowance (RDA) for iron is 18 mg/d for premenopausal women [43]. Most of the female amateur triathletes participating in this study, regardless of their type of diet (lacto-vegetarian, lacto-ovo-vegetarian, ovo-vegetarian, omnivorous, or pescatarian), had suboptimal intake of iron. It is possible that an imbalance between the consumption of plant-based foods, containing mainly less bioavailable non-heme iron, and the intake of meat, which not only contains well-absorbed dietary iron but also enhances non-heme iron absorption [44], may have compromised iron levels. Female endurance athletes are especially prone to iron deficiency because of iron losses associated with menstruation, elevated concentrations of hepcidin after strenuous exercises, foot-strike and intense training-related intravascular hemolysis, exercise-induced acute inflammation, sweating, as well as gastrointestinal bleeding [45,46,47]. Female athletes should, therefore, aim for a dietary iron intake above their RDA; that is, they should consume more than 18 mg of iron daily [29].

Calcium is a micronutrient necessary for bone mineralization. Inadequate dietary calcium intake reduces bone mineral density, thus placing calcium-deficient athletes at high risk for bone stress fractures during training and competition [48]. Studies indicate that high-intensity endurance training might enhance renal calcium excretion [49]. Nevertheless, there is no evidence that exercise-induced changes in metabolism increase calcium requirements [48]. Dietary calcium intake in premenopausal female athletes should be the same as recommended for the general population, i.e., 1000 mg/d [50,51]. However, LEA disturbs the hormonal balance and affects calcium and bone health [52,53]. Thus, a calcium intake of 1500 mg/d supplemented with 1500–2000 IU/d of vitamin D is recommended to optimize bone health in female athletes with LEA or menstrual dysfunction [29]. In our study, triathletes exhibiting symptoms related to LEA (from the H-LEA group) consumed the amount of calcium recommended for the general population; they failed to meet recommendations for dietary calcium intake for athletes with LEA. These participants also tended to consume more plant-based protein and fiber, which might impair intestinal calcium absorption and put them at risk of calcium deficiency, increasing their susceptibility to stress fractures and osteoporosis.

Food should provide an adequate energy intake, adjusted to maintain normal nutritional status. However, food components can influence gastrointestinal function, induce satiety, decrease food intake, and affect the energy density of the diet. Low-energy-density diets are associated with lower daily energy intake, placing female endurance athletes at risk of RED-S. As a high intake of fiber-rich and plant protein-based foods might decrease the energy density of the diet, female triathletes should reduce the dietary intake of whole-grain products and limit the intake of dried fruits and bran. They should also avoid foods rich in fiber and plant protein, such as legumes and peanuts, replacing them with tofu, dairy products, eggs, and meat. Female triathletes might benefit from an increased frequency of meals and diets that include more easily digestible foods and offer a balanced composition of fatty acids.

### Limitations

This study is not free of some limitations. The small sample size could have led to underestimating or overestimating the effects, thus limiting the generalizability of the results. However, we aimed to study top-performing female triathletes, so the study group size was relatively small. Future studies should validate these findings in a larger sample.

Another limitation of this study is the absence of direct body composition measurements; because of that, we could not estimate relative macronutrient intake or energy availability adjusted by fat-free mass. We calculated relative macronutrient intake adjusted by body mass.

Our study used wrist-worn activity trackers to estimate exercise energy expenditure. Such devices may have some drawbacks; for example, they may underestimate energy expenditure and introduce some variability into the readings. However, most of our study participants (except two) used wearables from the same brand (Garmin), thus reducing the potential risk of variability. An analysis of the behavior of free-living athletes using wrist-worn activity trackers often provides more ecologically valid and contextual information than an analysis conducted in a controlled laboratory setting.

## 5. Conclusions

This study found that in a sample of 20 top-performing female amateur triathletes, 30% were at risk of RED-S, and 50% showed at least one symptom related to LEA. Diets consumed by triathletes exhibiting menstrual dysfunction, gastrointestinal symptoms, and/or injuries, despite no differences in macronutrients energy intake, were higher in fiber, differed in protein type (more plant-sourced than animal-sourced), and the proportion of fatty acids (more unsaturated than saturated) from diets of triathletes without LEA symptoms, who ate more saturated fatty acids. Our findings suggest that diets high in fiber and plant-based protein can place highly trained female triathletes at risk of RED-S. They also indicate that dietary fat composition is essential for adequate energy intake, specifically for triathletes, as they demonstrate a greater reliance on free fatty acids as energy substrates.

## Figures and Tables

**Table 1:** Baseline characteristics of top-performing female amateur triathletes participating in the study.

Variable	Mean	SD
Age	37.8	9.00
Height [cm]	169.8	5.48
Body mass [kg]	62.3	7.72
BMI [kg/m^2^]	21.6	2.03
Training load [h/week] ^1^	11.0	3.76
Training session [min/d] ^2^	84.3	25.4

BMI, body mass index; ^1^ self-reported; ^2^ estimated using wrist-worn activity trackers.

**Table 2:** Diet, triathlon, and scores of the LEAF-Q in top-performing female amateur triathletes.

Participant	Diet	Triathlon *	LEAF-Q Injuries	LEAF-Q Gastrointestinal Symptoms	LEAF-Q Menstrual Function	LEAF-Q [pts]
1	OV	IM	4	5	3	12
2	OV	IM	2	6	1	9
3	OV	1/2 IM	0	0	7	7
4	OV	1/2 IM	1	4	12	17
5	OV	DIM	0	0	3	3
6	LA	1/2 IM	0	0	0	0
7	PE	IM	0	1	0	1
8	OV	1/2 IM	0	0	3	3
9	OV	DIM	2	2	0	4
10	OV	1/4 IM	0	0	1	1
11	LO	1/4 IM	0	2	4	6
12	OV	1/2 IM	0	6	0	6
13	LA	1/2 IM	0	3	0	0
14	LA	1/2 IM	4	3	4	11
15	OV	1/2 IM	0	5	0	5
16	OO	1/4 IM	0	0	0	0
17	OV	1/4 IM	0	0	0	0
18	OV	IM	0	1	7	8
19	OV	1/4 IM	2	0	4	6
20	OV	1/2 IM	0	3	5	8

* The longest triathlon race; BMI, body mass index; 1/4 IM, Quarter Ironman (swim 0.95 km, bicycle 45 km, run 10.5 km); 1/2 IM, Half Ironman, Triathlon 70.3 (swim 1.9 km, bicycle 90 km, run 21 km); IM, Ironman, Triathlon (swim 3.9 km, bicycle 181 km, run 42 km); DIM, Double Ironman (swim 7.6 km, bicycle 360 km, run 84.4 km). Diet: LA, lacto-vegetarian; LO, lacto-ovo-vegetarian; OO, ovo-vegetarian; OV, omnivorous; PE, pescatarian; LEAF-Q, Low Energy Availability in Females Questionnaire. Different colors are allocated to various LEAF-Q scores: LEAF-Q score ≤ 2 pts (blue), LEAF-Q score 3–5 pts (green), LEAF-Q score 6–7 pts (orange), LEAF-Q score 8–10 pts (red), and LEAF-Q score ≥ 11 pts (dark red).

**Table 3:** Resting energy expenditure, total energy expenditure, energy intake, and energy deficit in top-performing female amateur triathletes.

Variable	L-LEA *	H-LEA *	Cohen’s d	Effect Size	95% CI	*p* ^1^
REE [kcal]	1382.8 ± 94.4	1390 ± 87.9	0.079	negligible	−0.86, 1.02	0.8619
aTEE [kcal/d]	2436 ± 219	2532 ± 247	0.414	small	−0.54, 1.36	0.7302
rTEE [kcal/kg/d]	39.86 ± 4.37	40.49 ± 3.34	0.163	negligible	−0.78, 1.10	0.7205
aEI [kcal/d]	1946 ± 362	2204 ± 442	0.640	medium	−0.32, 1.60	0.1697
rEI [kcal/kg/d]	32.2 ± 7.88	35.45 ± 8.21	0.403	small	−0.55, 1.35	0.3793
aED [kcal/d]	−490.1 ± 456	−328.7 ± 492	0.340	small	−0.61, 1.29	0.4564
rED [kcal/kg/d]	−7.65 ± 7.23	−5.04 ± 8.05	0.341	small	−0.61, 1.29	0.4552
rED [%]	−19.24 ± 18.2	−12.26 ± 19.9	0.366	small	−0.58, 1.31	0.4234

REE, resting energy expenditure; aTEE, absolute total energy expenditure; rTEE, relative total energy expenditure; aEI, absolute energy intake; rEI, relative energy intake; aED, absolute energy deficit; rED, relative energy deficit; L-LEA, low LEA (LEAF-Q score 0–5 pts); H-LEA, high LEA (LEAF-Q score ≥ 6 pts); * mean ± SD; CI, confidence interval; ^1^ Student *t* test or Mann–Whitney test.

**Table 4:** Baseline characteristics, self-reported training load, and the training sessions estimated using wrist-worn activity trackers in top-performing female amateur triathletes from the L-LEA and H-LEA groups.

Variable	L-LEA *	H-LEA *	Cohen’s d	Effect Size	95% CI	*p* ^1^
Age	37.5 ± 7.04	38.1 ± 11.01	0.065	negligible	−0.87, 1.00	0.8862
Height [cm]	170.1 ± 6.42	169.5 ± 4.69	−0.107	negligible	−1.05, 0.83	0.8142
Body mass [kg]	61.7 ± 8.14	62.9 ± 7.67	0.152	negligible	−0.79, 1.09	0.7382
BMI [kg/m^2^]	21.24 ± 1.88	21.87 ± 2.23	0.306	small	−0.64, 1.25	0.5032
Training load [h/week] ^2^	12.5 ± 4.79	9.5 ± 1.35	−0.852	large	−1.83, 0.13	0.0374
Training session [min/d] ^3^	88.4 ± 33.5	80.2 ± 14.3	−0.318	small	−1.26, 0.62	0.7620

BMI, body mass index; L-LEA, low LEA (LEAF-Q score 0–5 pts); H-LEA, high LEA (LEAF-Q score ≥ 6 pts); * mean ± SD; CI, confidence interval; ^1^ Student *t* test or Mann–Whitney test; ^2^ self-reported; ^3^ estimated using wrist-worn activity trackers.

**Table 5:** Daily intake of fiber, proteins of plant origin, polyunsaturated fatty acids, monounsaturated fatty acids, and saturated fatty acids in top-performing female amateur triathletes.

Variable	L-LEA *	H-LEA *	Cohen’s d	Effect Size	95% CI	*p* ^1^
Fiber [g/d]	28.8 ± 7.2	33.5 ± 9.9	0.54	medium	−0.41, 1.50	0.2408
Plant PROT [g/d]	16.2 ± 4.4	26.6 ± 13.3	1.04	large	0.04, 2.05	0.0563
PUFA [g/d]	8.2 ± 2.2	12.2 ± 4.7	1.09	large	0.09, 2.10	0.0115
PUFAn6 [g/d]	3.9 ± 1.5	7.1 ± 4.1	1.02	large	0.03, 2.02	0.0329
PUFAn3 [g/d]	1.2 ± 0.7	1.6 ± 0.7	0.59	medium	−0.37, 1.55	0.2066
MUFA [g/d]	19.9 ± 9.2	28.0 ± 8.6	0.90	large	−0.08, 1.89	0.0590
SFA [g/d]	23.2 ± 13.6	19.6 ± 7.1	−0.33	small	−1.28, 0.61	0.4677

PPROT, proteins of plant origin; PUFA, polyunsaturated fatty acids; PUFAn6, *n*-6 polyunsaturated fatty acids; PUFAn3, *n*-3 polyunsaturated fatty acids; MUFA, monounsaturated fatty acids; SFA, saturated fatty acids; L-LEA, low LEA (LEAF-Q score 0–5 pts); H-LEA, high LEA (LEAF-Q score ≥ 6 pts); * mean ± SD; CI, confidence interval; ^1^ Student *t* test or Mann–Whitney test.

## Data Availability

The original contributions presented in this study are included in the article. Further inquiries can be directed to the corresponding author. The data are not publicly available as they contain information that could compromise the privacy of research participants.
